# I2DNet - Design and Real-Time Evaluation of Appearance-based gaze estimation system

**DOI:** 10.16910/jemr.14.4.2

**Published:** 2021-08-31

**Authors:** L R D Murthy, Siddhi Brahmbhatt, Somnath Arjun, Pradipta Biswas

**Affiliations:** 3D Lab, CPDM, Indian Institute of Science, Bangalore, India; Information Technology, G H Patel College of Engineering and Technology, India

**Keywords:** Eye tracking, web-cam based eye tracking, Convolutional Neural Networks, Usability evaluation

## Abstract

Gaze estimation problem can be addressed using either model-based or appearance-based approaches. Model-based approaches rely on features extracted from eye images to fit a 3D eye-ball model to obtain gaze point estimate while appearance-based methods attempt to directly map captured eye images to gaze point without any handcrafted features. Recently, availability of large datasets and novel deep learning techniques made appearance-based methods achieve superior accuracy than model-based approaches. However, many appearance- based gaze estimation systems perform well in within-dataset validation but fail to provide the same degree of accuracy in cross-dataset evaluation. Hence, it is still unclear how well the current state-of-the-art approaches perform in real-time in an interactive setting on unseen users. This paper proposes I2DNet, a novel architecture aimed to improve subject- independent gaze estimation accuracy that achieved a state-of-the-art 4.3 and 8.4 degree mean angle error on the MPIIGaze and RT-Gene datasets respectively. We have evaluated the proposed system as a gaze-controlled interface in real-time for a 9-block pointing and selection task and compared it with Webgazer.js and OpenFace 2.0. We have conducted a user study with 16 participants, and our proposed system reduces selection time and the number of missed selections statistically significantly compared to other two systems.

## Introduction

Eye gaze estimation has been an active research area for a long time due to its numerous applications. Early systems used techniques like electrooculography, whereas recent systems employed infrared imaging and computer vision techniques to obtain accurate gaze estimates. These infrared-based eye gaze trackers brought eye gaze tracking into the commercial realm and helped realize applications such as gaze-based human-computer interaction in automotive (32; 34), aviation (28) and assistive technology (3; 38) domains. Researchers also made progress to utilize gaze estimates for non-interactive purposes like visual scan path analysis (10 ), cognitive load estimation of drivers in automotive domain (29; 33). Recently, various efforts were being made to achieve similar performance using commodity hardware like built-in cameras present in laptops and smartphones as this can expand the reach of this technology. With the help of large datasets and advancements in deep-learning techniques, recent appearance-based approaches trumped model-based methods in terms of accuracy.

However, it was observed that appearance-based systems which reported state-of-the-art accuracy on one dataset do not achieve the same degree of accuracy on a different dataset. Spatial weights CNN model proposed by (48) reported 42 mm error on MPIIGaze dataset (47 ) whereas the same architecture reported 85.6 mm error on EYEDIAP dataset (16). Further, models which performed well during with-in dataset validation reported a higher error on cross-dataset validation. Diff-NN proposed in (26) used 9 reference samples and reported a 4.64° error on MPIIGaze during with-in dataset validation but reported an error of 9.8° mean angle error when trained on UT-Multiview (39) dataset and tested on MPIIGaze. A similar trend can be observed for the MeNet proposed by (43) which achieved 4.9° during with-in dataset validation but reported 9.51° error during the above mentioned cross-dataset validation setting. Researchers (26) believed that this is due to the discrepancies during pre-processing and variation in head pose and gaze data distributions. It may also be noted that both Diff-NN and MeNet utilized UT-Multiview dataset for training, which is collected in controlled lab conditions and tested on the MPIIGaze dataset, which is recorded in real-world conditions. Even though appearance variations across participants and inherent offset between visual axis and optical axis for each person do exist, high error during cross-dataset validation raises the question of how appearance-based approaches perform on unseen users under real-world conditions.

Appearance-based methods predict gaze angles in normalized space and transforming this to millimeters is not trivial as this also depends on the head pose of the user. Hence, most appearance-based systems reported their performance in terms of mean angle error, but not in pixels or millimeters. Thus, it is unclear how well the existing state-of-the-art appearance-based systems perform in an interactive context like an eye gaze controlled interface. (46) evaluated Spatial weights CNN in off-line mode under two lighting conditions for its accuracy and the evaluation presented in (18) focused on accuracy and latency. Both these evaluation works did not evaluate appearance-based systems in the context of interaction and usability.

Infra-red based commercial eye gaze trackers have much higher accuracy than current state-of-the-art appearance-based approaches. Yet, appearance-based gaze estimation systems have several use cases like webcam based gaze controlled interfaces as they do not require any additional hardware. Further, people with severe speech and motor impairment often use gaze controlled interface with limited number of screen elements (21; 38). Appearance-based gaze estimation systems can be used to build such gaze controlled interfaces on smartphones and tablet PCs using their front cameras.

This paper proposes a novel architecture that focuses on overcoming appearance-related variations across users to improve accuracy. We propose I2DNet: I-gaze estimation using dilated differential network which achieved a state-of-the-art 4.3 and 8.4 degree mean angle error during the evaluation on MPIIGaze and RT-Gene respectively. Further, to understand how the proposed system performs for unseen users in real-time, we conducted a user study. We compared its performance for a 9-block pointing and selection task with WebGazer.js (30 ) and OpenFace 2.0 (2).

This paper is structured as follows. The next section presents literature review of various gaze estimation methods and their evaluation methods. Section 3 and 4 present the methodology of our proposed model and experiments conducted on MPIIGaze and RT-Gene using our proposed architecture. Section 5 presents the design of our user study. Section 6 presents the results and analysis. Section 7 presents the discussion and future work followed by conclusion in section 8.

### Related work

In this section, we discussed various gaze estimation approaches followed by works which evaluated gaze tracking interfaces.

Infrared imaging-based eye trackers used feature-based methods for gaze estimation which extract features from the eye images. Numerous approaches were proposed for Point of Gaze (PoG) estimation for desktop and mobile settings based on the well-established theory of gaze estimation using the pupil centers and corneal reflections. Guestrin and Eizenman (19) reported to have obtained a PoG accuracy of 0.9° in a desktop setting based on an evaluation on 4 subjects. Further, Brousseau et al (4) proposed a system for gaze estimation for mobile devices compensating for the relative roll between the system and subject’s eyes. They evaluated their system on 4 subjects and reported around 1° of gaze estimation error. Even though these results were promising, it was unclear how these systems will perform on wider population under real-world usage conditions with wider gaze angles and head poses. Recent reports of the commercial IR-based gaze trackers claim to provide gaze accuracy of <1.9° error across 95% of population under real-world usage conditions (https://www.tobiidynavox.com/devices/eye-gaze-devices/pceye-mini-access-windows-control/#Specifications).

In addition to the desktop setting, head-mounted video-based eye trackers are becoming increasingly more popular. Morimoto and Mimica (27) stated that gaze estimation approaches based on pupil tracking techniques have better accuracy since they are not covered by eyelids. They reported that the pupil tracking based gaze estimation systems can achieve an accuracy of ~1°, but they also commented that it is hard to detect the pupil. Since then, several approaches like (13, 15; 36, 37) were proposed for robust real-time pupil detection in challenging natural environments like driving and walking. Current state-of-the-art approach (9) reported a pupil detection rate of ~85% on PupilNet (14) and LPW (42) datasets and a detection rate of ~74% on Swirski (40) dataset. Yet, the performance of these approaches in terms of gaze estimation in similar challenging environments with such pupil detection accuracies is still unknown.

Researchers also approached the problem of gaze estimation without using IR-illuminators. Such model-based approaches rely on the detection of visual features such as pupil, eyeball center and eye corners. These features are then used to fit a geometric model of the 3D eyeball to obtain eye gaze estimates. Early model-based methods such as (8 ), (1) and (22) relied on high-resolution cameras to detect such visual features with high accuracy, but these methods were not robust to variation in illumination conditions. Recent model-based methods like GazeML (31) and OpenFace 2.0 attempted to overcome these limitations by using only commodity web cameras and empowering their feature detectors using machine learning techniques. OpenFace 2.0 reported a state-of-the-art performance on the task of cross-dataset eye gaze estimation with an error of 9.1° on MPIIGaze. Webgazer.js proposed a feature-based method for gaze interaction for web-based platforms. The authors of Webgazer.js proposed to use left and right eye images to train a ridge-regression model and used cursor activity for fine-tuning of the predictions. They conducted an online evaluation with 82 participants and reported a best mean error of around 175 pixels across various tasks, which equates to around 35mm as per present display configurations. This error of 35mm is less than 42mm, which is achieved by Spatial weights CNN on MPIIGaze dataset. Even though we cannot make a direct comparison due to the different datasets used for evaluation, it may be noted that the WebGazer.js reported their performance across 82 participants while MPIIGaze dataset contains 15 participants.

In contrast to feature-based and model-based methods, appearance-based methods rely only on the images captured from off-the-shelf cameras and do not attempt to create handcrafted features from eye images. Instead, these methods utilize machine learning techniques to directly obtain the gaze estimates from eyes or face images. These appearance-based methods are strongly supported by the creation of large datasets and advancements in deep learning techniques. In terms of model architecture, appearance-based gaze estimation can be classified broadly into Multi-channel networks and Single-channel networks. We are aware of the gaze estimation datasets and approaches like Gaze 360 (23) which focused on 3D gaze estimation across 360° but for this literature review, we have focused on the approaches that worked on MPIIGaze, which is designed exclusively for laptop/desktop setting.

One of the first attempts of appearance-based gaze estimation was GazeNet (47), which is a single channel approach where a single eye image is used as the input to an architecture based on 16-layer VGG deep CNN. Head pose information was concatenated to the first fully connected layer after convolutional layers. GazeNet reported a 5.4° mean angle error on the evaluation subset of MPIIGaze, termed as MPIIGaze+. This work was followed by another single-channel approach, by (48) where full face images were provided as input instead of eye crops. Authors of this work used the spatial weights approach to provide more weightage to the regions of face which were significant for gaze estimation. (35) proposed a branched architecture where a single eye image and head pose were used with a switch condition imposed on the head pose branch.

As an alternative to single-channel approaches, numerous multi-channel approaches were proposed. (25) proposed one such multi-channel convolutional neural network called iTracker. They used left eye image, right eye image, face crop image and face grid information as inputs. The face grid contained the location of face in the captured image. Subsequent multi-channel approaches did not use face grid as input. Our work is closely related to Multi-Region Dilated-Net proposed in (5) which used dilated convolutions instead of several maxpooling layers in their CNN architecture. This approach also reported the same result of 4.8° mean angle error as (48) did on MPIIGaze+. As an extension of this work, they utilized gaze decomposition (6) in addition to dilated convolutions and achieved 4.5° error on MPIIGaze. Further, most recent work by (6) proposed face-based asymmetric regression-evaluation network which utilized the asymmetry between two eyes of same person to obtain gaze estimates. In this work, they evaluated the confidence score for gaze estimate obtained from each eye image and relied on the stronger prediction. This work presented two versions of the method: FARE Net and FAR-Net* which reported state-of-the-art performance of 4.41 and 4.3° error on MPIIGaze dataset, respectively.

As mentioned in the Introduction section, several of these approaches with high accuracy during within-dataset validation did not report same degree of accuracy under cross-dataset validation. Most of these cross-dataset validation experiments were conducted with UT-Multiview as training set and MPIIGaze as test set. It may be noted that the former was collected in controlled laboratory setting whereas the latter was recorded in real-world condition with variations in appearance, illumination, and inter-eye illumination difference. Due to the unavailability of such large datasets collected in real-world conditions apart from MPIIGaze, it is unclear how these models would perform under real world conditions when trained on MPIIGaze. Further, most of these works had little focus on the usability of these networks for real-time gaze interaction.

Zhang et al. (46) attempted to evaluate the network’s performance proposed in Zhang et al. (48) against another model based method GazeML and commercial Tobii EyeX eye tracker. They evaluated these three systems in off-line mode on 20 participants under two illumination conditions and at 8 different distances between user and the camera. They recorded 80 samples under each of these 16 conditions and used 60 of these as calibration samples for fine tuning the gaze predictions and reported accuracy on remaining 20 samples. They used third-order polynomial fitting to map 2D gaze predictions to actual screen coordinates. Even though this study attempted to study the accuracy of various gaze estimation approaches, no emphasis was made on the usability aspect. Further, fine tuning of network for each distance may not be applicable for practical applications.

Summarizing our literature review, we believe that there is still a lack of clarity regarding the performance of existing appearance-based gaze estimation models for day-to-day gaze interaction for unseen users and little evidence is available on their usability. We also believe that an architecture that is robust to appearance-related variations is imperative. In this direction, we propose a novel architecture that attempts to overcome appearance-based variations. Further, we have conducted one of the first real-time user study to evaluate the usability of an appearance-based gaze estimation system.

## I2DNet – Methodology

### Dilated Convolutions

Our proposed architecture contains mainly two components. The first part, inspired from (5) uses dilated convolutions to obtain larger receptive field instead of relying on several maxpooling layers. Appearance variations at eye regions when a person is gazing at two different screen locations might be subtle and the difference between these two eye images can be over tens of pixels as illustrated in the above mentioned work. Most current architectures use several downsampling layers like convolutional layer with large stride and pooling layers as we go deeper into the network. The use of maxpooling layers is common in tasks like object detection where maxpooling aims to achieve shift-invariance by reducing the resolution of feature maps (17 ). It also helps in increasing the effective receptive field, the region in the input space that each CNN feature is looking at. However progressively using maxpooling layers reduces the spatial resolution of feature maps. In other words, the subtle changes captured over the range of fewer pixels might be lost if we use maxpooling layers successively as these prevents passing of finer spatial information to deeper layers of the network.

Dilated convolutions achieve large receptive field without resorting to maxpooling layers. In simple terms, dilated convolution is a convolutional operation applied to an input with defined gaps in the kernel. For a 2D image, a dilated convolution with a dilation rate of 1 produces same output as a normal convolutional operation. Stacking convolutional layers increases receptive field linearly, whereas stacking dilated convolutions with varied dilation rate can increase receptive field exponentially. (5) showed that the use of dilated convolutions in place of normal convolutions improves gaze estimation using a multi-channel architecture.

We have not only used dilated convolutions for eye regions as Dilated-Net did, but also for the face channel since changes in facial expression brings appearance-related changes in eye region. We have also changed the number of filters in each layer of the network. Intuitively, deeper layers of network capture high level features and it is a common paradigm in most of the computer vision tasks to gradually increase the number of filters as we go deeper into the network. We have followed the same paradigm and have increased the number of filters across all three channels.

### Differential Layer

Our novel contribution to the multi-channel architecture of Dilated-Net is in terms of employing a differential layer that obtains the difference between features obtained from left and right eye channels. (44) demonstrated hierarchical nature of features learnt by the network with respect to layers in the network. They showed that shallow layers encode low level features like edge and color conjunctions whereas deeper layers represent entire objects with significant pose variation. From this understanding, we proposed the following approach.

First, we obtained feature maps for left and right eye images from the shared convolutional layers. These feature maps contained higher level features that encode information about various portions in both the eye images. These portions may include eyeball, sclera region or brow region and these vary from person to person. Since left and right eye images contain common features which may be redundant for gaze estimation, we investigated whether omitting these person-dependent common features improved gaze estimation accuracy. Hence, in order to retain features pertinent to gaze estimation and to remove redundant and person-specific features from learning process, we proposed to obtain difference of the left eye and right eye features. We trained our gaze estimator on the absolute difference of eye features along with the features obtained from face channel. Even though the face channel brings in person-specific features into the learning process, we preferred to retain it as it encodes head pose information which is important for gaze estimation. We posit that the resultant difference vector acts as a better feature transformation than the case where the features from both eyes were just concatenated for subsequent fully connected layers.

We hypothesize that the use of differential layer along with dilated convolutions improves gaze estimation performance than the results reported in (5). Note that our approach is fundamentally different from the Diff-NN proposed by (26). Their work considers two input images belonging to the same eye (left or right) and attempts to learn the gaze differences. Their approach focuses on person-specific gaze estimation whereas we rely on a total person-independent approach. Further their idea relies on the ability of the network to learn gaze differences given two eye images whereas we incorporated a differential layer into our network architecture which aims at circumventing the redundant, subject-specific features by leveraging left and right eye features to improve the person-independent gaze estimation. In the next section, we present experiments on MPIIGaze and RT-Gene datasets using our proposed approach.

## I2DNet – Experiments

### Datasets and Pre-processing

### MPIIGaze

We used the evaluation subset of the MPIIGaze dataset from (48 ) where 45,000 images with full faces were provided along with ground truth gaze points. MPIIGaze dataset was collected in real-world conditions with wide illumination and head pose variations and it is considered as a challenging dataset. It was collected with 15 people from diverse ethnic backgrounds and includes appearance-variations like wearing spectacles. We used the facial landmark annotations provided along with the dataset and applied image normalization based on the method proposed in (45). In simple terms, this method assumes a virtual camera with a focal length f_v_ and applies translation and rotational transformations on the image so that it faces the reference point from a distance d_v_ and cancels out the roll angle of the head. To obtain the normalized face images for our multi-channel network, we assumed the center of the face as the reference point. Similarly, for normalized eye images, respective eye center was considered as the reference point. Our normalization process performs grayscale conversion, perspective warping and histogram equalization in the same order. Similar process with different parameters was applied to obtain normalized left eye and right eye images. We obtained face crops of size 120x120 and eye crops of size 36x60 which are fed into the network. We performed preliminary experiments on MPIIGaze to determine optimal value of d_v_. We performed experiments for the values 400, 500 and 600 and found 600 to provide lower mean angle error when two subjects in the MPIIGaze dataset were used as test set. Hence, we used d_v_ as 600 for eye images and 1000 for face images. We used f_v_ as 960 for both face and eye images as prescribed in (45).

### RT-Gene

RT-Gene dataset contains 122,531 images of 15 participants using wearable eye tracking glasses. Unlike MPIIGaze dataset where participants sat near to their computers, participants here were located at 0.5 to 2.9 meters from the camera during this dataset creation. Compared to MPIIGaze, this dataset has wider variation in terms of head pose and gaze angles. Since this dataset uses wearable eye tracking glasses while capturing the images, they used semantic inpainting to paint the area covered by eye tracking glasses with appropriate skin texture. Hence, the authors provided both original and in-painted version of the images after normalizing them. The resolution of the normalized eye and face images provided is 36x60 and 224x224 respectively. We did not do any further processing of these images apart from resizing of face images to 120x120. We observed noises in the in-painted set as (7) reported and hence used only the original dataset for our experiments. We used grayscale images for all our experiments on both datasets.

### Testing Procedure and Results

**Figure 1.**
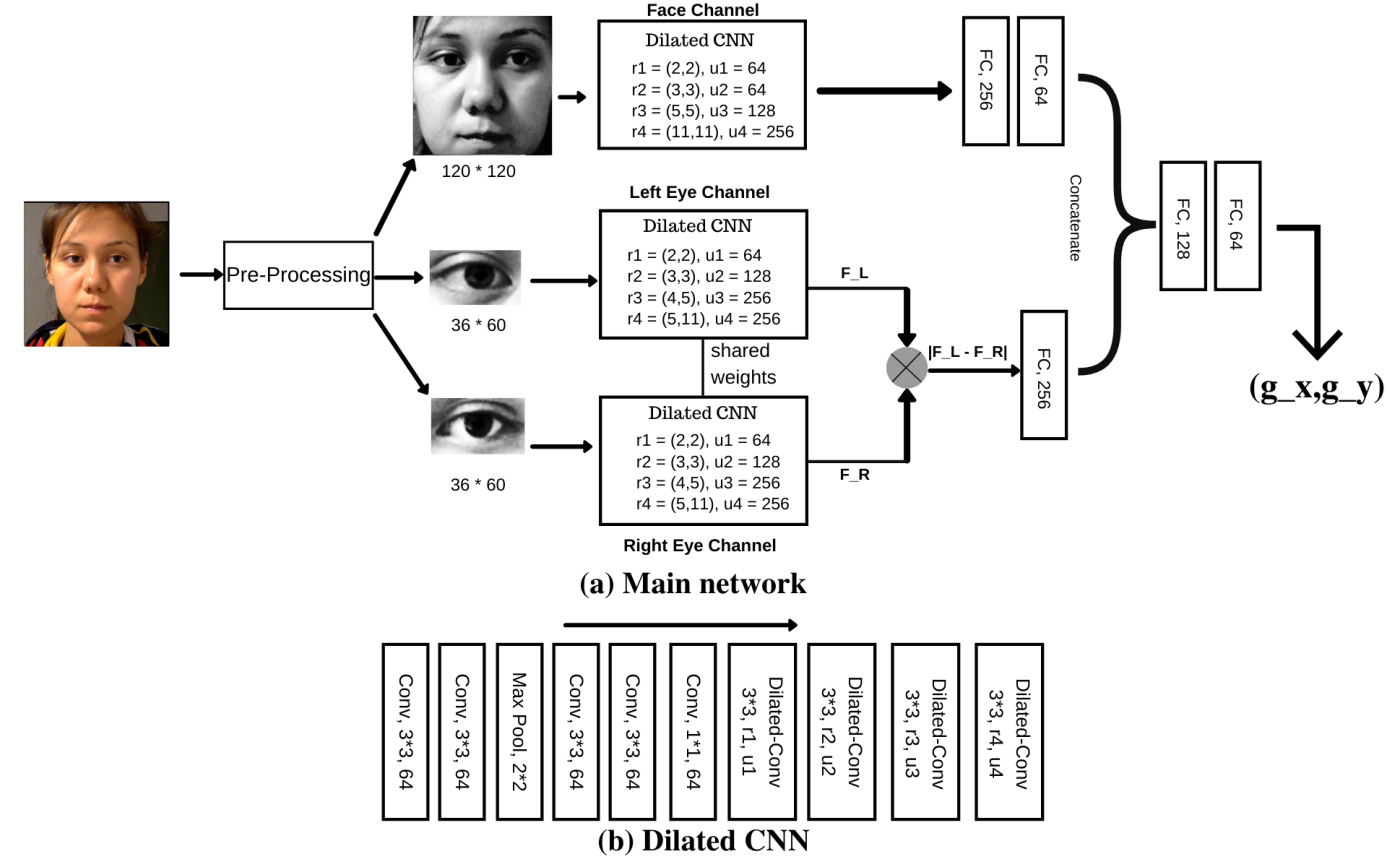
Architecture of I2DNet network. (a) Main network represents our architecture. (b) Dilated CNN represents the generic CNN block that we used across face and both eyes channels

We used the architecture detailed in figure 1 and conducted experiments on the normalized dataset. The parameters r1, r2, r3 and r4 in figure 1 represents the dilation rate for each layer where as u1, u2, u3 and u4 represents the number of filters in each layer. We undertook a series of experiments to fine tune the hyper-parameters of the network like dilation rates, number of feature maps, dropout values and kernel regularizers. We also undertook a series of experiments on MPIIGaze dataset similar to the studies to obtain the optimal d_v_ value for hyper-parameter tuning. We presented the optimum hyper-parameter values obtained from our experiments in figure 1. We followed similar procedure as (5; 26; 48 ) for cross-subject validation on MPIIGaze dataset. We conducted leave-one-out cross-validation on MPIIGaze. During each fold, we randomly chose 15% of the training data as validation set. g_x and g_y obtained at the end of the network in figure 1 represents the predicted pitch and yaw gaze angles in normalized space. Since the ground truth gaze angles are in radians and many of the values are less than 1, we used a scaling factor of 100 and we used mean squared error as the loss function. We used Adam optimizer (24) with a batch-size of 32. We computed cosine similarity between all predicted and ground truth gaze points using their 3D normal vector representations to obtain angle error.

We conducted experiments on RT-Gene dataset as per the evaluation protocol provided by the dataset. We divided the original dataset into 3 folds and we performed a 3-fold cross validation. We presented mean angle error of our experiments using the proposed model and compared it against various other approaches in Table 1. We achieved a state-of-the-art mean angle error of 4.3 ± 0.97 and 8.44 ± 1.08 degrees on MPIIGaze and RT-Gene datasets respectively, lower than both Spatial weights CNN and GEDDnet (6). We achieved on-par performance with FAR* Net. Note that the proposed model employs smaller number of parameters (~87M) than GEDDnet (~107M), Spatial weights CNN (~196M) and FAR* Net (~848M). We utilized around 10% of the trainable parameters compared to FAR* Net and yet achieved same degree of performance. We reported the results of iTracker as reported in (48 ) to make it comparable with other approaches as the results reported in original paper were in centimeters.

**Table 1:** Mean Angular Errors for Gaze Estimation

**Models**	**MPIIGaze**	**RT-Gene**	**# Parameters**
iTracker	6.2°	-	~8M
Spatial Weights CNN	4.8°	10.0°	~196M
RT-GENE (12)	4.8°		~30M
Dilated-Net	4.8°	-	~5 M
GEDDnet	4.5°	-	~107 M
FAR* Net	4.3°	8.4°	~848 M
RT-GENE (4 ensemble)	4.3°	8.6°	~122M
**I2DNet (Proposed)**	**4.3**°	**8.4**°	**~87 M**

### Ablation Study

Further, to illustrate the effect of our differential layer on the gaze estimation accuracy, we performed ablation study on the same CNN-backbone without the differential layer using MPIIGaze dataset. We removed the differential layer illustrated in Figure 1 and concatenated the left and right eye feature vector along with face feature vector for gaze estimation. We obtained a mean angle error of 4.54° on MPIIGaze. It may be noted that we achieved this result using only dilated convolutions and it is on par with GEDDnet, which relied on both dilated convolutions and gaze decomposition. Hence, using our ablation study, we demonstrated that the presence of differential layer indeed improves gaze estimation accuracy from 4.54° to 4.3°. Further, we noted that the presence of difference layer reduces the number of parameters by ~32K and yet improved the gaze estimation accuracy.

**Figure 2.**
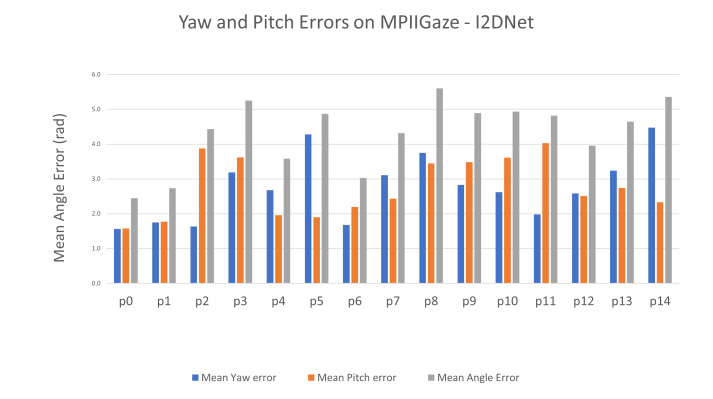
Mean Angle Errors for all participants in MPIIGaze using I2DNet.

In figure 2, we plot mean angle error for each participant from MPIIGaze dataset in cross-validation setting and the corresponding yaw and pitch error components. We computed difference between predicted and ground truth gaze points in their 2D normalized angular representations to obtain yaw and pitch errors. We observed that even though there is no clear pattern among pitch and yaw error components across participants, 8 out of 15 participants displayed higher pitch error than yaw error.

Hence with our proposed approach, we showed a 10% improvement in terms of gaze estimation error over our baseline (5) (4.3° vs 4.8°). We undertook a paired t-test which revealed that our proposed approach performed statistically significantly better (t[14] = 2.17, p=0.047, Cohen’s d=0.5) than the baseline (5). We further evaluated our model in real-time interactive setting. Our user study design is explained in the next section.

## Design of user study for gaze controlled interface

We investigated and analyzed the performance of our proposed I2DNet in terms of angle error on MPIIGaze and RT-Gene datasets. Since we wanted to study its performance in an interactive setting like a gaze controlled interface, we evaluated the proposed system on a 9-block selection task (38). From our literature review, OpenFace, a model-based approach, reported least cross-dataset validation error of 9.1° on MPIIGaze, lower than other appearance-based approaches like Diff-NN or MeNet. Further, as we mentioned earlier, WebGazer.js reported an average error of 175 pixels i.e., ~35 mm in a user study which involved 82 participants. Hence, we compared the proposed system’s performance with OpenFace and WebGazer.js.

### Task

**Figure 3.**
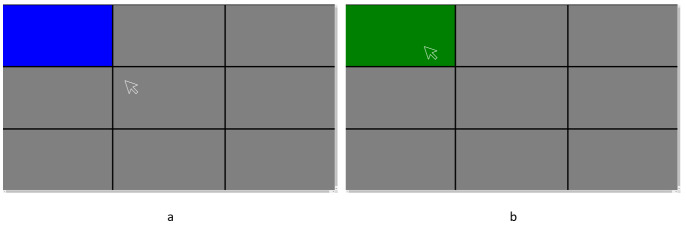
(a) Task screen with blue stimuli (b) When user makes selection, the blue block turns green.

**Figure 4.**
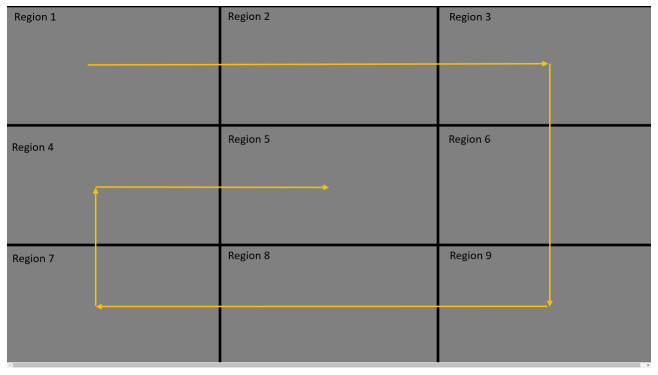
Region annotations for 9 blocks on screen

We divided the entire screen into 9 blocks of equal area. At first, we displayed these nine blocks on screen in gray color. We mapped the above mentioned three gaze prediction systems’ outputs to a marker on screen. As illustrated in figure 3a, we provided a stimulus to the user by randomly changing one of these nine blocks to blue color. The user was instructed to fixate attention at the block whichever turns blue and press space bar on keyboard. The blue block turned green when the user pressed spacebar while the marker was present inside its boundary as illustrated in figure 3b. We defined “*selection time*” as the time between the instance a block turned blue and the instance the block turned green. If the user could not make selection with in 10000 milliseconds, we counted it as “*miss click”* and stimulus was moved on to a different block. Figure 4 represents the annotations for the 9 blocks which we shall use for the rest of our paper.

### WebGazer.js

We setup WebGazer.js software using the provided library at this hyperlink. We enabled Kalman filter provided along with the library to filter noise present in gaze predictions and to make the trajectory of predicted points smooth. This system required user to calibrate before they can start interacting. The calibration step required user to click on 9 dots, 5 times on each placed at different locations on screen. WebGazer.js reported that their system self-calibrates using the clicks and cursor movement. At the end of calibration process, WebGazer.js asked users to stare at a point and reported calibration accuracy. Participants who obtained low calibration accuracy had to repeat the calibration process. We set the minimum value of calibration accuracy as 80% for the participant to proceed to the task. We mapped gaze predictions to a red marker as discussed in previous section. For clear visibility of the marker, we set its size same as the mouse cursor. We ensured that the participants’ head lies in the pre-defined bounding box prescribed by WebGazer for proper tracking throughout the experiment.

### OpenFace

During our preliminary studies, 3 participants used OpenFace 2.0 and reported off-set between actual gaze and cursor position with noticeable noise. They also said that they could not reach certain portions of the screen. Hence, we designed a custom calibration routine based on smooth pursuit principle. We asked users to follow a circle which traverses across the screen. We recorded gaze predictions from OpenFace during the smooth pursuit. We trained a classifier network which took gaze angles as inputs and block prediction the user is gazing at as the output.

The circle moved from top-left block (Region 1) to left bottom block (Region 7) through right top block (Region 3) and right bottom block (Region 9). From left bottom block, the circle reached the center block of screen (Region 5). We represented this trajectory in figure 4 in yellow lines. Throughout its trajectory, the circle moved at constant pace and halted at the center of each of these nine blocks for 2 seconds. Our calibration routine received these gaze angle predictions from OpenFace through UDP socket. We accounted for latency caused by both system and user and prepared our training data accordingly.

**Figure 5.**
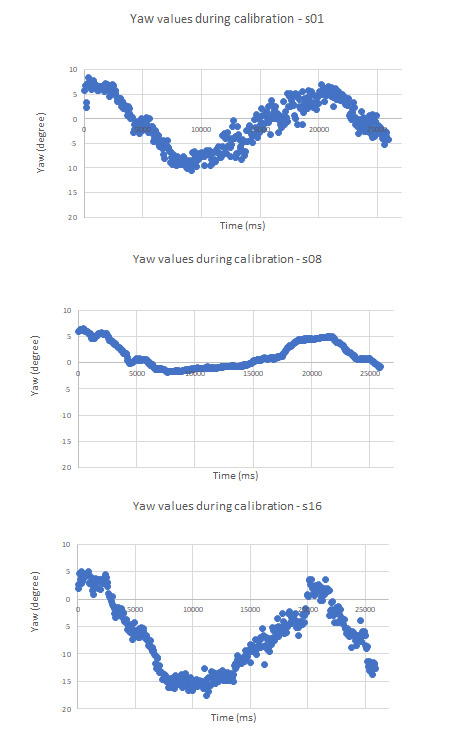
Profile of yaw predictions by OpenFace while users performing our calibration routine.

Figure 5 illustrates the raw yaw predictions obtained from OpenFace when three of our participants undertook the above explained calibration routine during the pilot study. The scatter plot indicated that the raw yaw predictions contained noise. We observed difference in minimum and maximum yaw values among participants for the same gaze positions on screen. Hence our calibration routine not only maps gaze predictions to respective blocks, but it also smoothens the noise present in the predictions. We used a 3-layer fully connected neural network which takes gaze yaw and pitch as inputs and predicts one of the 9 blocks as output. Similar to WebGazer.js, we monitored the classification accuracy during training. Participants were allowed to proceed for the task only if the classification accuracy on test set exceeded 75%. We observed that the classification accuracy for participants who used only eye movements with their head fixed was poor. Hence, we suggested our users to use head movement along with eye movement while using this interface. The mouse cursor is placed at the center of the predicted block. We named this interface which uses OpenFace 2.0 and our calibration procedure as OpenFace_NN and used the same for the rest of the paper.

### I2DNet - Tracker

We used the same architecture presented in section 4. For real-time evaluation, we trained the network using the entire MPIIGaze dataset. To ensure proper training and to prevent overfitting, we utilized 15% of the dataset as validation set and observed training and validation losses over the training process of 25 epochs.

For each frame from webcam, we obtained 3D facial landmarks and head pose using OpenFace 2.0. We used these 3D landmarks of two corners of both eyes and two mouth corner points to obtain face center. In the same fashion, we used 3D landmarks of two corners of each eye to obtain corresponding eye center. We used these centers, d_s_ and f_v_ and the process explained in previous section to obtain normalized face and eye images. These images were fed to the trained I2DNet to obtain gaze predictions. We computed the screen dimensions, camera intrinsic parameters and performed extrinsic camera calibration to obtain screen-camera pose using the method described in (41 ). For this purpose, we captured the images of checkerboard pattern displayed on screen by the webcam using a planar mirror. Using these images, we performed the screen-camera pose estimation. We obtained gaze vector from I2DNet and face center, face rotation matrix from OpenFace for each frame captured through webcam. We then used these metrics to obtain gaze point on screen using the method described in (46). For our proposed system, we did not utilize any calibration or filtering techniques, rather we directly map the predicted gaze points on screen to mouse cursor. We named our real-time gaze interface built using I2DNet predictions as I2DNet-tracker.

### Experiment Design

We used repeated measures approach and enrolled 16 users to participate in our user study (Age range from 19 to 51 years). Out of 16, 4 were female and 7 users wore spectacles while performing the task. We conducted our experiment in a closed room under artificial illumination. We used MSI GE75 Raider 9SG laptop with intel-i7 processor, GeForce RTX 2080 graphics card and a webcam of 1280x720 resolution. We used the same resolution for all the three systems. No user had any exposure to eye tracking technology prior to our experiment. Users performed the task elaborated in section 5.1 using three systems. Under each trial, each user got 25 stimuli and hence we recorded 1200 (25x3x16) selections in total and 400 selections using each system. We randomized the order of three systems for each participant to minimize the learning effect. We instructed users to perform the selection as soon as they can. In this experiment, we recorded selection time for each click and block locations for miss clicks. We did not pose any limitations on the head pose user can have during the task. Each of these systems may have various degrees of error for different users. Hence, we instructed users that systems may contain offset, and they can look anywhere inside the blue-colored block. After each session, we instructed users to answer the NASA TLX and SUS questionnaires for qualitative estimation of perceived cognitive load and system usability. We also recorded subjective feedback apart from these questionnaires.

## Results and analysis

### Summary of Quantitative and Qualitative Results

**Figure 6.**
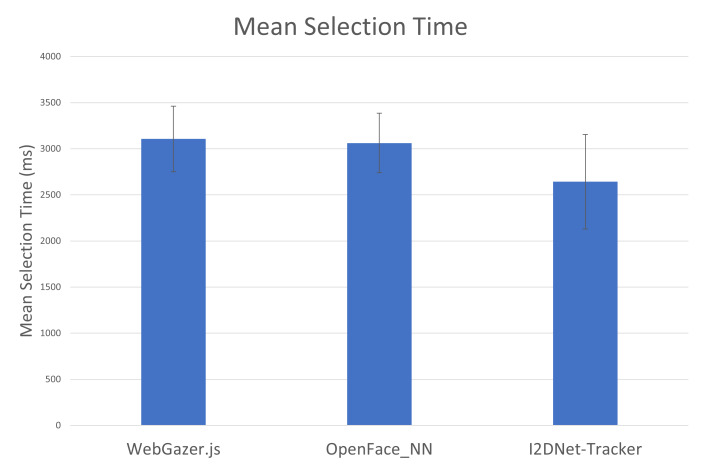
Mean Selection time using three gaze prediction systems.

In this section, we presented the quantitative and qualitative results of our user study. Figure 6 showed the mean selection time averaged over all participants for three interfaces. We undertook one-way ANOVA for selection times but did not find any significant effect. We conducted three pairwise t-tests and found that participants can perform the task significantly faster (p<0.05) using I2DNet-Tracker (2.6 sec) than WebGazer.js (3.1 sec).

**Figure 7.**
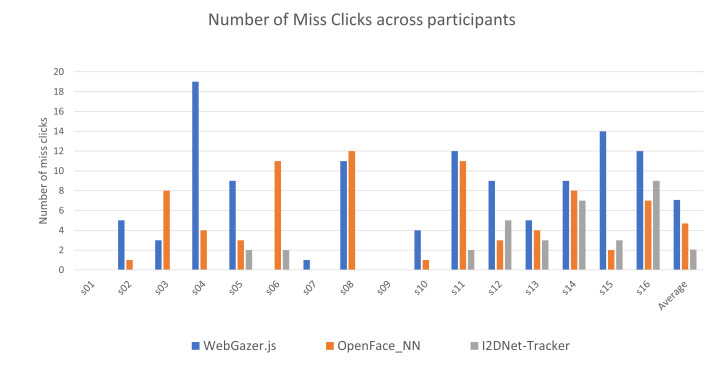
Number of miss clicks for all participants in our user study.

The other two pairwise t-tests did not show any significant effect. Figure 7 represents the number of miss clicks for each participant while using three interfaces. We undertook one-way ANOVA for number of miss clicks as well and found significant difference among three interfaces F (2,45) = 5.16, p<0.05, η^2^ = 0.186.

Three pairwise t-tests found that participants missed significantly lesser number (p<0.05) of stimuli using I2DNet-tracker (33 miss clicks) than both WebGazer.js (113 miss clicks) and OpenFace_NN (75 miss clicks) systems. The other pairwise t-test between OpenFace_NN and WebGazer.js did not show any significant effect. Extending these results, we observed that the success rate of users to perform the designed task using I2DNet-tracker, OpenFace_NN and WebGazer.js was

91.75%, 81.25% and 71.75% respectively. Further, we noted that three participants recorded 21 miss clicks (63.6% of the total) using I2DNet-tracker. Further, eight participants did not record any miss clicks while using I2DNet-tracker.

We also analyzed qualitative metrics collected during our user study. In Table 2, we summarized the mean NASA TLX score and SUS score for all the three interfaces. A one-way ANOVA test did not find significant effect for both TLX and SUS scores. On summarizing three pair-wise t-tests performed on TLX scores, we inferred that the participants perceived significantly lesser cognitive load while using I2DNet-tracker compared to WebGazer.js. Further, three pair-wise t-tests on SUS scores showed that the subjective preference to I2DNet-Tracker was significantly higher than to the WebGazer interface. Even though TLX score and SUS score was favorable to OpenFace_NN compared to WebGazer.js, a pair-wise t-test did not show the statistical significance.

**Table 2:** Qualitative metrics of our user study

**Interface**	**TLX Score**	**SUS Score**
WebGazer.js	37.4	72.6
OpenFace_NN	32.0	75.5
I2DNet-Tracker	**26.5**	**81.8**

### Comparison between participants with and without spectacles

To understand how usage of spectacles impact the performance of the three eye gaze tracking systems under consideration, we divided our 16 users into two groups based on their use of spectacles. As mentioned earlier, s02, s05, s06, s08, s13, s14 and s16 were our 7 participants who used spectacles. The diopter rating for these participants ranged from -1 to -5. As a result, the 9 participants who did not use spectacles were classified as “Group A” and the rest were classified as “Group B”. In Table 3, we summarized the average selection time and miss clicks for these two groups.

Across the interfaces, participants with spectacles missed higher number of stimuli than the participants without spectacles. WebGazer.js did not show significant difference between these two groups in terms of mis clicks, but interestingly participants without spectacles (Group A) took more time to select the stimulus block. Participants in both groups took similar time while using OpenFace_NN interface, but participants with spectacles missed more stimuli. In case of I2DNet-tracker, the effect of presence of spectacles was evident since both average

**Table 3:** Selection Time and Miss clicks comparison between participants with and without spectacles

**Interface**	**Group A**		**Group B**	
	**Selection Time (ms)**	**Miss Clicks**	**Selection Time (ms)**	**Miss Clicks**
WebGazer.js	3271	6.8	2893	7.3
OpenFace_NN	3083	3.2	3035	6.5
I2DNet-Tracker	2373	1.1	2988	3.2

selection time and average missed clicks were higher in Group B. It may be noted that the I2DNet-tracker interface does not use any calibration prior to the task whereas both other methods used calibration procedures either for mapping or to fine-tune the gaze predictions specific to user. These results are in-line with the results reported on similar analysis presented in (46), where appearance-based systems outperformed model-based system in overall accuracy, but a significant difference among these two groups while using appearance-based systems persists.

### Miss Clicks – Region-wise Analysis

**Figure 8.**
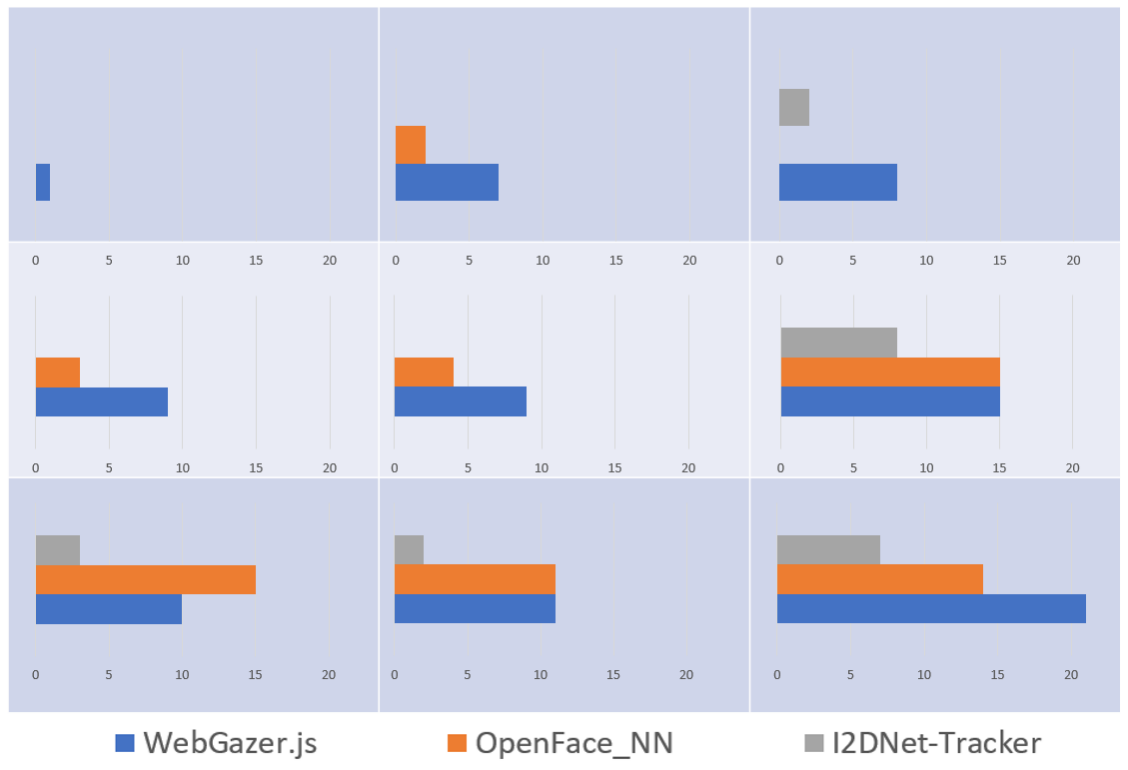
Region-wise miss clicks using three interfaces across the screen.

Next, we analyzed the regions of miss clicks occurred on screen across the interfaces. Figure 8 contains 9 regions representing our 9 blocks on screen. The bar graphs indicate the number of miss clicks occurred in each region while using three interfaces. The scale on x-axis is maintained the same across all 9 regions. It can be inferred that participants recorded miss clicks across all regions of screen while using WebGazer.js. While using OpenFace_NN, significant miss clicks were observed from Region 6 onwards. A significant portion (~69%) of miss clicks using I2DNet-tracker occurred in Region 6 and Region 9, the right and bottom extremes of the screen. This can be partially explained using figure 2, where higher pitch error was observed in higher number of participants in MPIIGaze experiments.

### Pointing and Selection Task Results on 4x4 Grid

We further conducted evaluation for finer level of 16-block selection task on our system. We recruited 5 participants, one with spectacles and the rest without wearing spectacles. Each participant performed 30 selections using our system and hence in total, 150 selections were recorded on the 4x4 grid. We observed similar results as that of 9-block task for these participants with mean selection time of 1.5 seconds. The only participant with spectacles recorded one miss click while the rest of them recorded none.

## Discussion and Future work

We aimed to achieve similar degree of gaze estimation accuracy during both with-in dataset validations and real-world usage conditions. In this regard, we proposed I2DNet that aimed to circumvent any appearance-related artifacts in appearance-based gaze estimation task which hinders the generalization ability of the network.

Our I2DNet achieved 4.3° and 8.4° mean angle error on MPIIGaze and RT-Gene datasets respectively. We retained face channel which brings in significant appearance-related artifacts into the learning process. We did not feed head pose information into the network separately as the head pose information obtained during real-time using OpenFace 2.0 reported a mean error of 3° for head orientation. We shall investigate whether the present error obtained is due to the inherent offset between visual axis and optical axis of the individual or due to any other appearance-related or illumination-related factors. Further, we shall carry investigation on the effect of accuracy and latency with reduced size of face images.

We conducted our evaluation in a single illumination condition. We believed that such real-time evaluations of state-of-the-art models needs to be performed in various illumination conditions to comprehensively understand the usability of such systems in real-time. Even though OpenFace 2.0 reported state-of-the-art cross validation accuracy on MPIIGaze dataset, its precision is poor as illustrated in figure 5. As demonstrated in (11), precision of gaze estimates also need to be studied along with accuracy since poor precision significantly affects the usability. (46) used third-order polynomial fitting to adapt the gaze predictions for each participant. Studies on efficacy of such calibration and filtering techniques applied on gaze-predictions in the context of appearance-based estimation during real-time is also imperative.

We observed that more stimuli could not be selected during our study when they appeared in Region 6 and Region 9. We inferred that this might be due to the occlusion of eye region with brows while gazing bottom portions of the screen. Further work needs to be done to overcome this ubiquitous challenge as most of the commercially available laptops contain web cameras above the display. We plan to conduct evaluation for finer level of tasks like 25-block selection task to understand the breaking point of such systems. Further investigations are yet to be performed under various illumination conditions for 16 and 25-block selection task. We believe that such evaluations are not only critical to understand the limitations of these systems but also to understand the bounds of usability.

Even though our evaluation indicated superiority of our approach both quantitatively and qualitatively over other two methods, we noted that the other two methods can run on CPU alone while our method requires a GPU. We evaluated our method on a laptop with i5 CPU alone and found that the prediction rate is around 3 fps which is lower than other two systems. This requirement of GPU is an inherent requirement for all appearance-based gaze estimation systems, yet it is a limitation when compared to feature-based methods. We intend to overcome this by applying principles of dark knowledge (20) to train a smaller and faster network with a minimal loss in accuracy as (25 ) did to achieve real-time performance on a mobile device.

## Conclusion

In this paper, we presented I2DNet, an appearance-based eye gaze estimation system which used dilated convolutions and a differential layer to remove common redundant features present in left and right eye images to improve accuracy of gaze predictions. We conducted experiments on MPIIGaze and RT-Gene datasets and obtained a state-of-the-art mean angular error of 4.3° and 8.4° respectively, which is on par with FAR* Net, but with lesser parameters. We conducted one of the first real-time user study on an appearance-based gaze estimation system and evaluated the proposed system on an eye gaze controlled interface with 9-block selection task and compared its performance with WebGazer.js and OpenFace 2.0. Despite not performing subject-specific calibration, the proposed system outperformed other two systems in terms of both selection times and number of miss clicks. We analyzed our user study results and we noted that I2DNet-tracker system performed better for participants without spectacles than participants who used spectacles. We observed that participants missed stimuli in more regions of the screen and fewer number of participants recorded no miss clicks while using other two interfaces compared to the proposed system. Since the proposed system does not depend on any calibration routine, we foresee to deploy the proposed system to develop gaze controlled interface for the use of physically challenged persons.

### Ethics and Conflict of Interest

The author(s) declare(s) that the contents of the article are in agreement with the ethics described in http://biblio.unibe.ch/portale/elibrary/BOP/jemr/ethics.html and that there is no conflict of interest regarding the publication of this paper.

### Acknowledgements

We wish to thank all the participants who took part in the user study.
